# Hybrid Circuit of Memristor and Complementary Metal-Oxide-Semiconductor for Defect-Tolerant Spatial Pooling with Boost-Factor Adjustment

**DOI:** 10.3390/ma12132122

**Published:** 2019-07-01

**Authors:** Tien Van Nguyen, Khoa Van Pham, Kyeong-Sik Min

**Affiliations:** School of Electrical Engineering, Kookmin University, Seoul 02707, Korea

**Keywords:** memristor-CMOS hybrid circuit, defect-tolerant spatial pooling, boost-factor adjustment, memristor crossbar, neuromorphic hardware

## Abstract

Hierarchical Temporal Memory (HTM) has been known as a software framework to model the brain’s neocortical operation. However, mimicking the brain’s neocortical operation by not software but hardware is more desirable, because the hardware can not only describe the neocortical operation, but can also employ the brain’s architectural advantages. To develop a hybrid circuit of memristor and Complementary Metal-Oxide-Semiconductor (CMOS) for realizing HTM’s spatial pooler (SP) by hardware, memristor defects such as stuck-at-faults and variations should be considered. For solving the defect problem, we first show that the boost-factor adjustment can make HTM’s SP defect-tolerant, because the false activation of defective columns are suppressed. Second, we propose a memristor-CMOS hybrid circuit with the boost-factor adjustment to realize this defect-tolerant SP by hardware. The proposed circuit does not rely on the conventional defect-aware mapping scheme, which cannot avoid the false activation of defective columns. For the Modified subset of National Institute of Standards and Technology (MNIST) vectors, the boost-factor adjusted crossbar with defects = 10% shows a rate loss of only ~0.6%, compared to the ideal crossbar with defects = 0%. On the contrary, the defect-aware mapping without the boost-factor adjustment demonstrates a significant rate loss of ~21.0%. The energy overhead of the boost-factor adjustment is only ~0.05% of the programming energy of memristor synapse crossbar.

## 1. Introduction

The human brain’s neocortex covers the brain’s surficial area, which is known to carry out the most intelligence functions. The thickness of neocortex has been observed as thin as 2.5 mm, where six layers are stacked one-by-one [1,2,3]. The six neocortical layers seem to be columnar, in which the complicated vertical and horizontal synaptic connections are intertwined among neurons to form the 3-dimensional neuronal architecture [4,5]. The neocortical neurons collectively respond to human’s sensory information from retina, cochlea, and olfactory organ [6]. The collective activation of neocortical neurons are trained over and over with respect to time, by changing the synaptic connection’s strength according to the sensory stimuli. The neuronal activation and synaptic plasticity can be thought of as a fundamental aspect of human perception and cognition, which are computed in a different way from the conventional Von Neumann machines.

As a software framework, Hierarchical Temporal Memory (HTM) has been developed to model the cognitive functions of neocortex [7,8,9,10,11]. By doing so, HTM can recognize and interpret various spatiotemporal patterns, mimicking how the human brain’s neocortex understands human’s sensory stimuli. The software framework of HTM is divided into two functional blocks: Spatial Pooler (SP) and Temporal Memory (TM). The role of SP is receiving and learning the sensory information. In SP, the sensory information is transformed into the collective activation of neocortical neurons. From the biological experiments, the neocortical neurons have been observed to be activated sparsely, not densely, in response to human sensory stimuli. The sparse activation of neocortical neurons is mathematically described as Sparse Distributed Representation (SDR) in HTM [1]. After SP learning the spatial features of the sensory stimuli, TM responds to the temporal sequences of SDR patterns generated from SP. By learning the temporal sequences of SDR patterns, TM can perform recognition and prediction for them.

**Figure 1:**
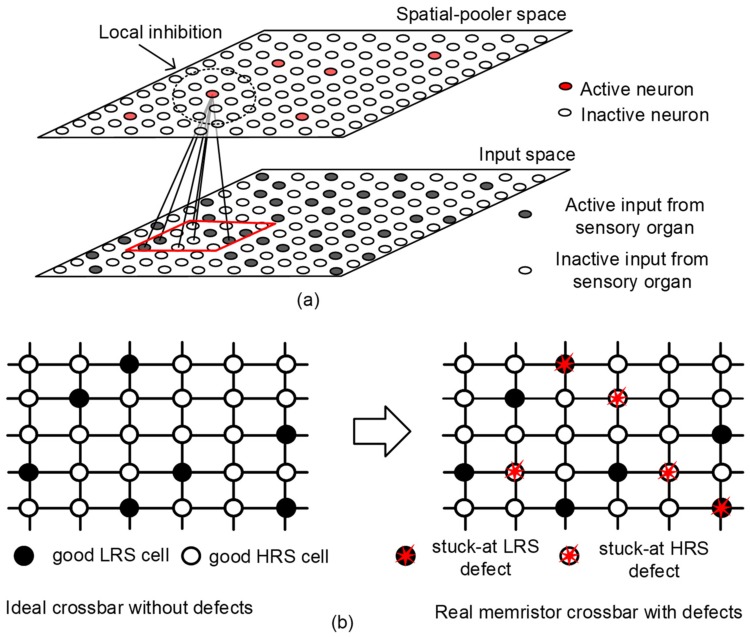
(**a**) The conceptual diagram of Spatial Pooler (SP) operation, where the input-space neurons are mapped to the SP neurons; and (**b**) the comparison of the ideal crossbar without defects and the real crossbar with defects. LRS and HRS mean Low Resistance State and High Resistance State, respectively.

Figure 1a shows a conceptual diagram of SP operation, where the input-space neurons are mapped to the SP neurons [8]. Here, the input-space and SP neurons refer to the neurons of sensory organ and neocortex, respectively. The sensory stimuli generated from the input-space neurons are connected with the neocortical neurons, as indicated in Figure 1a. The lines between the input and the SP spaces represent the synaptic connections. Synaptic weights of the connections are trained according to Hebbian learning rule in HTM [8]. If an SP neuron becomes active, in response to an input-space stimulus, the synaptic weights belonging to this neuron are strengthened, and weakened otherwise [8]. The circle zone in the SP space represents a local inhibition area, within which only few neurons are allowed to be active. In HTM, the size of inhibition zone in the SP space can be decided to control the sparsity of neuronal activation. It has been known that the percentage of neuronal activation is as sparse as 2% on average in the brain’s neocortex. This low sparsity of neuronal activation may have something to do with high energy-efficiency of neocortical cognitive operation.

In the previous publications, we developed hybrid CMOS-memristor circuits for implementing HTM, which was developed as the software framework originally, as mentioned earlier [12,13]. Memristors have been studied intensively for many years for their potential in neuromorphic hardware, since the first experimental demonstration [14,15]. This is because the memristive behaviors seem very similar with the experimental synaptic plasticity observed from biological neurons. From the biological experiments, the synaptic connections have been observed to be strengthened or weakened dynamically by electrical spiking signals applied to them [16].

Moreover, memristors can be fabricated to build 3-dimensional crossbar architecture using the CMOS-compatible Back-End-Of-Line (BEOL) process [17,18]. The 3-dimensional connectivity of memristor-synapses is very similar to the anatomical structure of the biological neocortex. In terms of cognitive functions, the memristor crossbar can perform vector-matrix multiplications in parallel, which can be considered very important in implementing energy-efficient computing like human brain’s cognition, unlike the state-of-the-art Von Neumann based computers [19,20].

One important thing to consider in the memristor crossbar is defects, as shown in Figure 1b. In the real memristor crossbar, there are stuck-defects, such as stuck-at-0, stuck-at-1, etc. [21]. In addition, variation-related defects can also be considered, where each memristor can have different LRS and HRS values due to process variations [22]. Here, LRS and HRS mean Low Resistance State and High Resistance State, respectively. Figure 1b compares the ideal crossbar (without defects) and the real one (with defects). The solid and open red circles with stars represent stuck-at-LRS and stuck-at-HRS defects, respectively. For the memristor defects such as stuck-at-faults and variations, these defects may be caused from the random nature of filamentary current path which can be formed or erased by the applied current and voltage to the memristor. The filamentary current path created or erased during the memristor programming can have statistical distributions like FLASH memory. Various statistical distributions by device-to-device, wafer-to-wafer, lot-to-lot, and process-to-process lead to the variations in memristance and stuck-at-faults [21].

To minimize a loss of recognition rate due to these memristor defects, we can consider the defect-tolerance scheme based on the conventional defect-aware mapping [21]. To explain the previous defect mapping scheme, the following logic function is assumed, $f=X_{1}X_{2}+X_{2}X_{3}+X_{3}X_{1}+/X_{1}/X_{2}/X_{3}$ is implemented in the crossbar [21]. 

**Figure 2:**
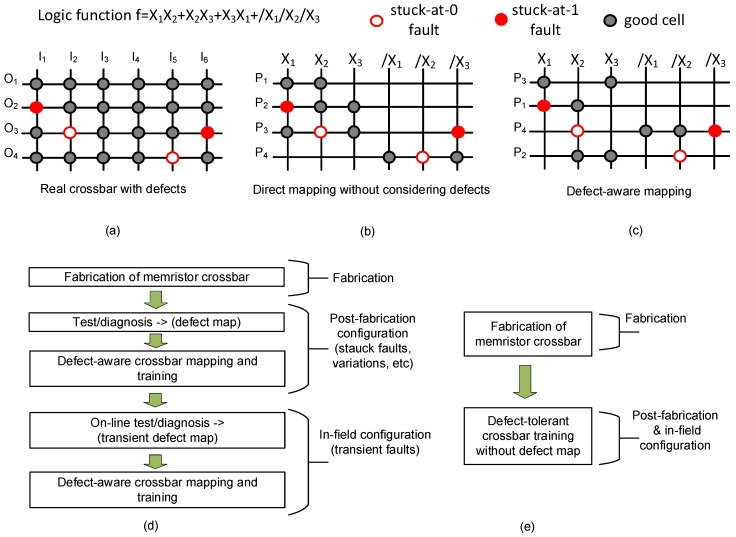
(**a**) The real crossbar with defects; (**b**) the direct mapping of the logic function without considering the defect map; (**c**) the defect-aware mapping of the logic function with considering the defect type and location; (**d**) the flowchart of crossbar training using the conventional defect-aware mapping [21]; and (**e**) the proposed flowchart of the defect-tolerant crossbar training without using the defect map.

In the logic function, /X_1_ means the inversion of X_1_. Figure 2a shows the real memristor crossbar (with defects). Here, I_1_, I_2_, etc. represent input columns. O_1_, O_2_, etc. are output rows. The gray circle indicates a good memristor cell, which can be programmed with HRS or LRS. The solid and open red circles represent stuck-at-1 and stuck-at-0 defects, respectively. Figure 2b shows the direct mapping without considering the defect map. P_1_, P_2_, P_3_, and P_4_ indicate the first, second, third, and fourth partial products in the target logic function. P_1_ calculates X_1_X_2_. However, P_2_ calculates X_1_X_2_X_3_, not X_2_X_3_ defined in the logic function, because of the stuck-at-1 fault on the crossing point between X_1_ and P_2_. P_4_ also calculates the wrong partial product. The stuck-at-0 fault is found at the crossing point between /X_2_ and P_4_. By doing so, P_4_ calculates /X_1_/X_3_ instead of the target product of /X_1_/X_2_/X_3_.

Figure 2c shows the defect-aware mapping, where the defects can be used in implementing the logic function according to the defect type and location. To do so, the crossbar’s rows in Figure 2c are reordered to consider the defect type and location in calculating the partial products. For example, the first row in Figure 2c is assigned to P_3_, not P_1_. P_1_ is assigned to the second row to calculate X_1_X_2_. The stuck-at-1 fault on the second row can be used in calculating P_1_ = X_1_X_2_. Similarly, the stuck-at-1 fault on P_4_ can be employed to calculate P_4_ = /X_1_/X_2_/X_3_. Moreover, the stuck-at-0 faults on P_2_ and P_4_ do not cause a wrong result for the calculation of partial products of P_2_ and P_4_. As shown in Figure 2c, the defects can be employed in implementing the target logic function according to the defect type and location. However, the defect-aware mapping scheme demands very complicated circuits, such as memory, processor, controller, etc., to be implemented in hardware.

Figure 2d shows the flowchart of crossbar training using the conventional defect-aware mapping. After fabricating the memristor crossbar, the defect map should be obtained by measuring the crossbar. As a post-fabrication configuration, the trained synaptic weighs can be transferred to the crossbar using the defect-aware mapping, as explained in Figure 2c. To do so, however, the complicated digital circuits, such as memory, controller, processor, etc., are needed for implementing the defect-aware mapping in hardware, as mentioned earlier.

Not using the defect-aware mapping, in this paper, we propose a simple memristor-CMOS hybrid circuit of defect-tolerant spatial-pooling, which does not need the complicated circuits of memory, controller, processor, etc., as shown in Figure 2e, where, unlike in Figure 2d, the crossbar’s defect map is not used. For developing the hybrid circuit of memristor-CMOS, we first show that the spatial-pooling based on Hebbian learning can be defect-tolerant, owing to the boost-factor adjustment, in Section 2. Additionally, we propose a new memristor-CMOS hybrid circuit, where the winner-take-all circuit is implemented not using capacitors occupying large area. In Section 3, the proposed hybrid circuit is verified to be able to recognize well Modified subset of National Institute of Standards and Technology (MNIST) hand-written digits, in spite of memristor defects such as stuck-at-faults, variations, etc. In Section 4, we discuss and compare the following three cases: (1) Spatial-pooling without both the boost-factor adjustment and the defect-aware mapping, (2) spatial-pooling with the defect-aware mapping, and (3) spatial pooling with the boost-factor adjustment, in terms of hardware implementation, energy consumption, and recognition rate. Finally, in Section 5, we summarize this paper.

## 2. Materials and Methods

To develop a memristor-CMOS hybrid circuit for realizing HTM’s SP function by hardware, memristor defects such as stuck-at-faults and variations should be considered. To consider the memristor defects in developing the hybrid circuit of the SP function, we explain the memristor fabrication and its behavioral model in the following sub-section of ‘a. Materials’. Then, we describe the boost-factor adjustment in HTM’s SP operation can make it defect-tolerant, because the false activation of defective columns in the crossbar are suppressed, in the sub-section of ‘b. Methods (scheme)’. In the sub-section of ‘c. Method (circuit)’, we propose the memristor-CMOS hybrid circuit by explaining its schematic and operation in detail. The hybrid circuit with the boost-factor adjustment is discussed and compared with the previous techniques without the boost-factor adjustment, later in this paper. The simulation result and comparison indicates that the memristor-CMOS hybrid circuit with the boost-factor adjustment can improve the recognition rate by more than ~20%, than the previous defect-map-based technique. This hybrid circuit can be very useful for energy-efficient computing in future IoT systems, where many IoT sensors are connected to a cloud of centralized data processing, as explained later.

**a. Materials**

**Figure 3:**
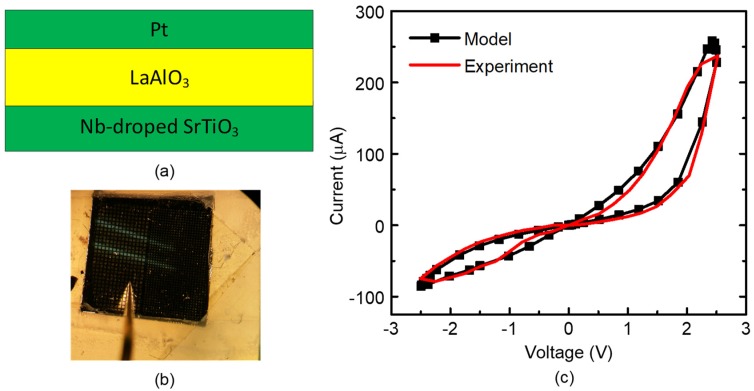
(**a**) The cross-sectional view of the measured memristor [23]; (**b**) the microscope picture of the measured memristor [24]; and (**c**) the memristor’s current–voltage relationships of the measurement and Verilog-A model [23].

Figure 3a shows a cross-sectional view of the fabricated memristor in this paper. The fabricated memristor has a film structure made of a Pt/LaAlO_3_/Nb-doped SrTiO_3_ stacked layer [23]. A microscope picture of the measured device is shown in Figure 3b, where the top electrode area is 100 µm × 100 µm [24]. The top and bottom electrodes were formed by Platinum (Pt) and SrTiO3, in the measured device, respectively [23].

Figure 3c shows current–voltage relationships of the fabricated memristor and the Verilog-A model, respectively [23]. The measurement was performed by Keithley-4200 (Semiconductor Characterization System, Tektronix, Inc., Beaverton, OR, USA) [23]. Here, the HRS/LRS ratio in Figure 3c was observed as large as 100. The black and red lines in Figure 3c represent the behavioral model of memristors and the measured data, respectively. The behavioral model described by Verilog-A in Figure 3c was used in the circuit simulation of the memristor-CMOS hybrid circuit in this paper.

**b. Methods (scheme): boost-factor adjustment scheme for defect-tolerant spatial-pooling**

**Figure 4:**
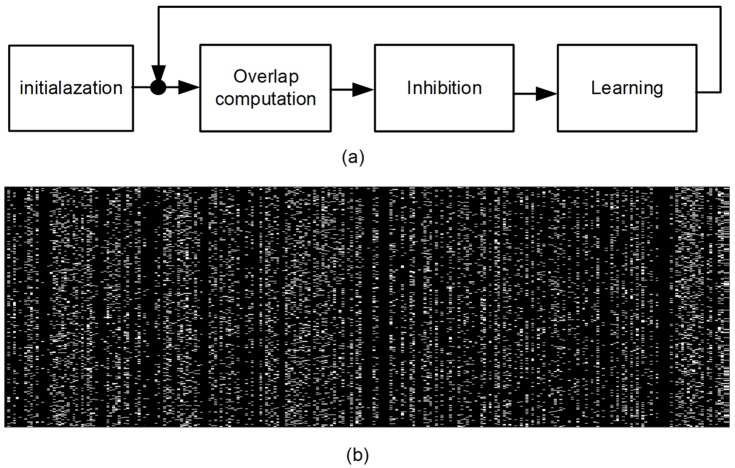
(**a**) The spatial-pooling algorithm composed of initialization, overlap computation, inhibition, and learning [8,12]; and (**b**) the defect map of a memristor crossbar with 10% random defects. Here the numbers of rows and columns of the crossbar are 400 and 256, respectively. The random defects are stuck-at-LRS and stuck-HRS defects.

The spatial-pooling in HTM software framework is composed of initialization, overlap computation, inhibition, and learning, as shown in Figure 4a [8,12]. After the initialization step (Phase 1), three steps: Overlap computation (Phase 2), inhibition (Phase 3), and learning (Phase 4), are repeated sequentially [8,12]. In Phase 1, random sets of inputs are selected from the input space, as indicated in Figure 1a. The number of random sets of inputs per training vector is the same with the number of crossbar’s columns. Each input in this random set can be connected to an output neuron in the SP via a synapse [8,12]. In Phase 2, an amount of overlap of each output neuron with the chosen set of inputs from the input space is calculated [8,12] The amount of overlap of each neuron in the SP can be calculated with the number of the connected synapses with the active inputs, multiplied by each column’s boost factor. In Phase 3, we decide which columns can be winners within the inhibition radius [8,12]. By doing so, the sparsity regarding the percentage of activation in the neocortical neurons can be controlled to not exceed a certain limit. In the case of the human brain’s neocortex, only 2% of neocortical neurons have been observed to be activated in response to human sensory stimuli. In Phase 4, Hebbian learning is performed to strengthen and weaken synaptic connections [8,12]. For the winners chosen in Phase 3, the synaptic permanence values for the active inputs are increased by p+. For the inactivate inputs, the permanence values are decreased by p-. p+ and p- represent the increment and decrement of synaptic permanence, respectively. The permanence value is allowed to vary between 0 and 1. If it reaches 1 or 0, the synaptic weight is changed to LRS or HRS, respectively.

One more parameter needed to be updated after the activation of each neuron is a boost factor. The boost factor can be defined with the following inverse relationship with the activity ratio [8]:

(1) $$b_{i}=e^{-\beta \left(a_{i}-\langle a_{i,neighbor}\rangle \right)}$$

Here, b_i_ means the boost factor of column, i. β is a positive parameter that controls the strength of the adaptation effect. a_i_ is the activity ratio of column i, and < a_i, neighbor_ > means the average activity ratio of the column’s neighborhood. For given M test vectors, the activity ratio of column, i, can be calculated with

(2) $$a_{i}=\frac{1}{M}\overset{M}{\underset{j=1}{\sum }}\left(activation\left(column,i\right)\right).$$

Here, a_i_ is the activity ratio of column i. M is the number of test vectors. The activation function defined with ‘activation(column, i)’ in Equation (2) becomes one, if the column, i, is activated. If the column is not activated, the activation function should be zero. For a neuron activated very frequently, its boost factor should be adjusted to be very small to lower the probability of activation. On the contrary, if a neuron is chosen very rarely, its boost factor should be increased. As explained just earlier, by adjusting each column’s boost factor according to each column’s activity ratio, the number of activations can be distributed more evenly for all columns in the crossbar.

We now discuss how each column’s activity ratio can be affected by memristor defects. Figure 4b shows a defect map of 400 × 256 memristor crossbar. Here, we assume random defects = 10% in the crossbar. The random defects can be stuck-at-HRS and stuck-at-LRS. Because the HRS defects do not cause erroneous activation of neurons, we focus on the LRS defects here.

**Figure 5:**
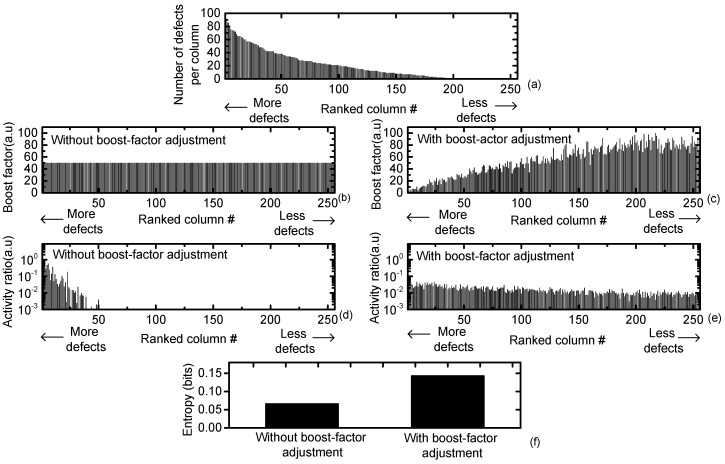
(**a**) The number of defects per column ranked from largest (left) to smallest (right); (**b**) the simulated boost factor of the crossbar without the boost-factor adjustment; (**c**) the simulated boost factor of the crossbar with the boost-factor adjustment; (**d**) the simulated activity ratio of the crossbar without the boost-factor adjustment; (**e**) the simulated activity ratio of the crossbar with the boost-factor adjustment; and (**f**) the comparison of crossbar entropy without and with the boost-factor adjustment.

In Figure 5a, the number of defects per column is ranked from the largest to the smallest. The number of columns in the crossbar is assumed to be 256 in Figure 5a. Each column is assumed to have 400 cells. Among the 400 cells per column, the most defective column has almost ~90 defects. The smallest number of defects per column is ~0. Figure 5b and c compare the simulated boost factors of the crossbars without and with the boost-factor adjustment, respectively. In Figure 5b, all 256 columns have the same boost factor, fixed by 50. On the contrary, in Figure 5c, each column’s boost factor is adjusted between 0 and 100, according to each column’s activity ratio.

Figure 5d and e compare the activity ratios of the crossbars without and with the boost-factor adjustment. Here, each column’s activity ratio is shown on the y-axis with respect to the ranked column number according to the number of defects. Figure 5d clearly indicates that a large number of defects in a defective column causes frequent activation of the column. The small number of defects results in the rare activation of the column. The frequent activation due to the defective column is very likely to be false and should be suppressed not to happen. Figure 5e shows that the frequent activation of the defective columns can be suppressed, by decreasing the boost factor of the defective columns lower than the neighbors. By doing so, we can reduce the false activation of the defective columns. Thus, the recognition rate loss due to the defective columns can be minimized by the boost-factor adjustment. 

In Figure 5f, the crossbar’s entropy is compared without and with the boost-factor adjustment. The entropy of the crossbar with N columns is calculated with Equation (3) [8].

(3) $$\mathrm{Entropy}=\overset{N}{\underset{i=1}{\sum }}\left[-a_{i}\log_{2}a_{i}-\left(1-a_{i}\right)\log_{2}\left(1-a_{i}\right)\right]$$

In Equation (3), ‘Entropy’ means the calculated amount of entropy. N is the number of columns in the crossbar. a_i_ is the activity ratio of column i. ‘log’ means the logarithmic function. Figure 5f indicates that the crossbar with the boost-factor adjustment shows much larger entropy than the crossbar without the boost-factor adjustment. The better entropy can result in a better recognition rate, as shown later in this paper.

**c. Methods (circuit): memristor-CMOS hybrid circuit of defect-tolerant spatial pooling**

**Figure 6:**
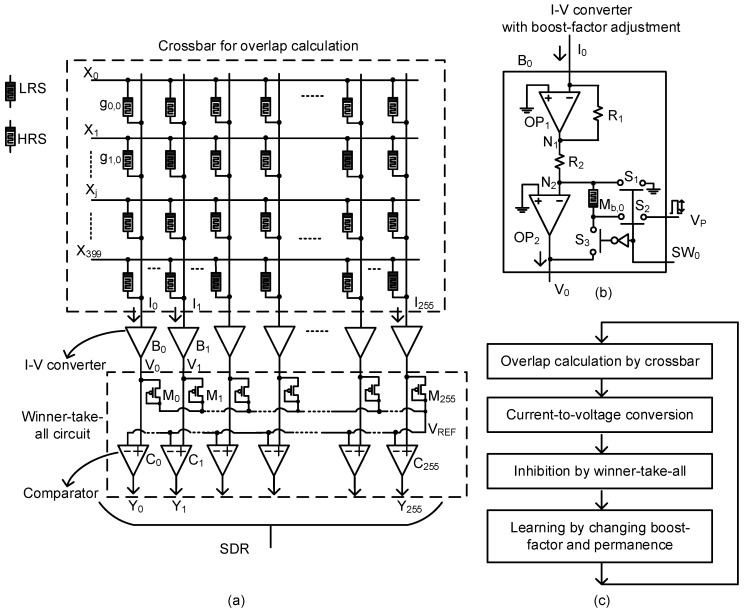
(**a**) The detailed schematic of the memristor-CMOS (Complementary Metal-Oxide-Semiconductor) hybrid circuit of defect-tolerant spatial pooling. The hybrid circuit is composed of the memristor crossbar, the current–to-voltage (I–to-V) converters with the boost-factor adjustment, and the winner-take-all circuit with the diode-connected Metal-Oxide-Semiconductor Field-Effect Transistors (MOSFETs) and comparators; (**b**) the detailed schematic of the voltage-converter circuit with the boost-factor adjustment; and (**c**) the operational diagram of the memristor-CMOS hybrid circuit.

Figure 6a shows a schematic of the memristor-CMOS hybrid circuit of defect-tolerant spatial pooling, where each column’s boost factor can be adjusted to make each column’s activity ratio more even. The memristor crossbar is composed of 400 rows and 256 columns for recognizing the MNIST hand-written digits. The 400 rows can receive 20 × 20 input pixels of each MNIST test vector. The 256 columns correspond to the 256 output neurons of the SP. In Figure 6a, X_0_ and X_1_ are the first and second row, respectively. g_0,0_ means memristor’s conductance of row #0 and column #0. Similarly, g_1,0_ means memristor’s conductance of row, #1, and column, #0. I_0_ and I_1_ represent the currents of columns, #0 and #1, respectively. I_0_ and I_1_ enter the current–voltage converters of B_0_ and B_1_, respectively, where each column’s boost factor can be adjusted according to each column’s activity ratio. Here, V_0_ and V_1_ are the converted voltages of columns #0 and #1, respectively. The converted voltages, V_0_ and V_1_, enter the comparators of C_0_ and C_1_, respectively, where V_0_ and V_1_ are compared with V_REF_. V_REF_ is obtained from the maximum output voltage among the neighbors using the diode-connected MOSFETs of M_0_, M_1_, etc. If we assume the diode ‘ON’ voltage is very small, V_REF_ can be very similar with the maximum voltage among all the output voltages such as V_0_, V_1_, etc. If V_0_ or V_1_ is very close to V_REF_, then column #0 or column #1 will be activated as a winner, inhibiting the neighboring columns from being activated. One thing to note here is that the V_REF_ for selecting the winner columns is obtained dynamically by extracting the largest output voltage among the neighbors. If a new input vector is applied to the crossbar, the output voltages are changed, too. Thus, we can obtain a new maximum voltage for the new input vector dynamically. Comparing each column’s output voltage with the new maximum, we can choose the next winner columns that are very close to the new maximum voltage. Y_0_ and Y_1_ refer to the output SDR bits for columns #0 and #1, respectively.

The circuit proposed in this paper does not use any capacitor for realizing the winner-take-all function, as shown in Figure 6a. This is different from the previous publications [12,20], where the capacitor was used to integrate the column current over time to accumulate the charge. The accumulated charge can be represented by the capacitor’s voltage. If one column’s voltage reaches a certain level at the earliest time, then that column is chosen as the winner [12,20]. Instead of capacitors occupying a very large area, the diode-connected MOSFETs are used here to obtain the maximum voltage among all output voltages, as shown in Figure 6a. The winning column can be chosen by comparing each column’s output voltage with the maximum voltage extracted from the diode-connected MOSFETs. The diode-connected MOSFETs in Figure 6a can occupy a much smaller area than the capacitors used in the previous publications [12,20].

One problem with the winner-take-all circuit using the diode-connected MOSFETs in Figure 6a is that the winner may be multiple, not single, in some cases. To investigate the number of winning columns per input vector, the statistical sparsity distribution is compared between the previous winner-take-all and the proposed circuit in Figure 6a. To do so, the average and variance of sparsity distribution are calculated for the previous winner-take-all and the proposed circuit in Figure 6a. The average sparsity of the previous winner-take-all and the proposed circuit in Figure 6a, are 2.2% and 2.3%, respectively. The calculated variance values are 0.09 and 0.11, respectively. The small difference in variance between the previous and proposed indicates that the winner-take-all can be implemented with the diode-connected MOSFETs and voltage comparators, not using the capacitors occupying a very large area.

Figure 6b shows a detailed schematic of the current-to-voltage converter, B_0_, with the boost-factor adjustment. OP_1_ and OP_2_ are OP amplifiers. The column current, I_0_, goes though R_1_. The node voltage, N_1_, becomes–I_0_ × R_1_. The converted voltage from I_0_ is given to R_2_. Thereby, the current though R_2_ goes to M_b,0_ and is finally converted to V_0_. V_0_ is the output voltage of the current–voltage converter. Here, we used R_1_ = 5 kΩ and R_2_ = 100 MΩ, respectively. For the boost-factor adjustment, M_b,0_ should be changed according to the activity ratio of column #0, with respect to the activity ratios of the neighbors. S_1_, S_2_, and S_3_ are the switches controlled by SW_0_. SW_0_ is applied by the boost-factor adjustment controller. V_P_ means the memristor programming pulse. V_P_ is applied to M_b,0_, through S_1_ and S_2_, to change the memristor’s conductance. V_P_ is applied to the boost-factor memristor for the boost-factor adjustment, when SW_0_ is high. On the contrary, when SW_0_ is low, S_3_ becomes ‘ON’ and V_0_ is compared with the other output voltages such as V_1_, V_2_, etc.

Figure 6c shows the operational diagram of the proposed memristor-CMOS hybrid circuit illustrated in Figure 6a,b. As indicated in Figure 6c, the crossbar performs the overlap calculation, in which the input voltage is multiplied with the memristor’s conductance. Then, each column’s current can be calculated by summating all the cell currents belonging to the column. The column current enters the current-to-voltage converter. The converted voltage from each column is delivered to the winner-take-all, where the winning column is chosen. Based on the winning column, the learning controller adjusts each column’s boost factor and the permanence values of the cells belonging to the column, according to Hebbian rule. The steps indicated in the operational diagram in Figure 6c are repeated again, when a new input vector is applied to the crossbar.

**Figure 7:**
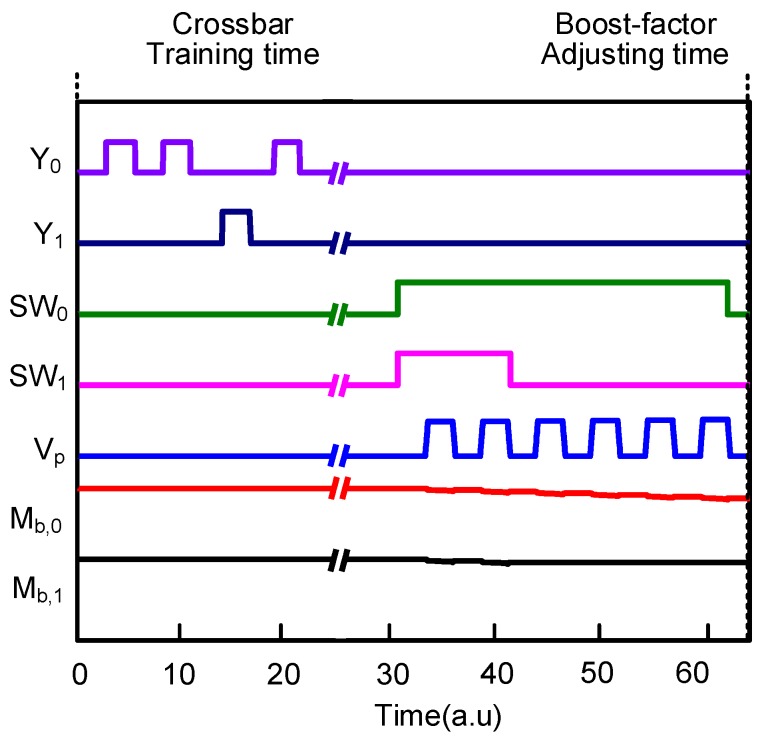
The simulated waveforms of the proposed memristor-CMOS hybrid circuit with the boost-factor adjustment.

The simulated waveforms in Figure 7 demonstrate the operation of the proposed memristor-CMOS hybrid circuit with the boost-factor adjustment. Here, the circuit simulation was performed using CADENCE SPECTRE (Cadence Design Systems, Inc., San Jose, CA, USA) and SAMSUNG 0.13-µm circuit simulation parameters [25]. The mathematical equations of the Verilog-A model of memristors used in the circuit simulation were explained in-detail in a previous publication [23]. In the simulation, we assumed the memristor crossbar of SP with 400 rows and 256 columns. The number of synaptic memristors per column is 25 among 400 cells. It means the 25 cells can be activated at the maximum among 400 cells per column in the crossbar. The increment and decrement of permanence are +0.01 and −0.01, respectively. The initial permanence values are assumed to be random between 0 and 1. The minimum amount of overlap between the input-space and spatial-pooler space can start from zero. The amount of overlap can be calculated by multiplying the input voltages with the memristor synaptic weights. The size of inhibition circuit zone is 64 columns in the crossbar. The number of winning columns is allowed not to exceed 2 among 64 columns. Thereby, the sparsity in Figure 6a can be controlled within 2%, as the brain’s neocortex does.

In Figure 7, during the crossbar training time, each column’s activity ratio is calculated by counting the number of activation of each column. If column #0 becomes activated, Y_0_ becomes high. Similarly, if column #1 becomes activated, Y_1_ is high. After the crossbar training time, the boost factor can be adjusted according to each column’s activity ratio, as described in Equation (1). The more frequent activation of column #0 leads to decrease the boost factor more. The less frequent activation of column #1 reduces the boost factor little, as shown in Figure 7. For adjusting the boost factors of columns #0 and #1, the pulse widths of SW_0_ and SW_1_, respectively, are modulated. M_b,0_ can be decreased more than M_b,1_ by many programming pulses of V_P_, because SW_0_ is high for a longer time than SW_1_. On the contrary, M_b,1_ is changed little, due to the fact that SW_1_ is high only for a very short time. The pulse modulation of SW_0_ and SW_1_ can be controlled very easily by counting the number of activations of each column during the crossbar training time.

## 3. Simulation Results

**Figure 8:**
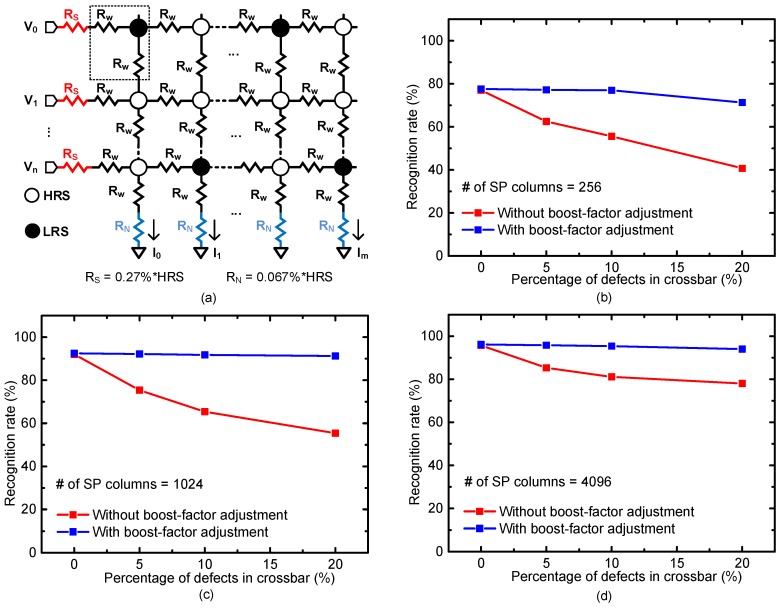
(**a**) The memristor crossbar with the non-ideal effects of R_S_, R_N_, and R_W_. Here R_S_ = 0.27%*HRS and R_N_ = 0.067%*HRS.; (**b**) the MNIST recognition rate of the non-ideal crossbar with 256 SP columns. Here, SP means Spatial Pooler. The percentage σ of memristance variation of HRS and LRS is assumed to be 0% in Figure 8.; (**c**) the MNIST recognition rate of the non-ideal crossbar with 1024 SP columns. Here the percentage σ of memristance variation of HRS and LRS is 0%; and (**d**) the MNIST recognition rate of the non-ideal crossbar with 4096 SP columns. The percentage σ of memristance variation in HRS and LRS is assumed 0%.

For calculating the recognition rate, we tested MNIST vectors [26,27] with the proposed memristor-CMOS hybrid circuit. To reflect the real crossbar with non-ideal effects, we considered source resistance, neuron resistance, wire resistance, etc., in the recognition-rate simulation [22]. Figure 8a shows a schematic of memristor crossbar that includes these non-ideal parasitic effects. Here, R_S_ and R_N_ represent source resistance and neuron resistance, respectively [2]. R_W_ represents wire resistance from metal layers. In the non-ideal crossbar, R_N_ and R_S_ are assumed to be 0.27% of HRS and 0.067% of HRS, respectively [22]. R_W_ is assumed to be ~1Ω per cell in this paper. These R_S_ and R_N_, which are 0.27% of HRS and 0.067% of HRS, respectively, are the worst-case values of the source and neuron resistance observed from the fabricated real crossbars [22]. In Figure 8a, V_0_, V_1_, and V_n_ represent the input voltages. I_0_, I_1_, and I_m_ represent the column currents. 

We now explain the crossbar architecture for recognizing the MNIST vectors. Here, the number of rows in the crossbar should be 400, which should be the same with the number of input voltages. Each MNIST vector is composed of 20 × 20 = 400 pixels. Thus, the number of input voltages is 400 for recognizing the MNIST vector. For the number of columns of the SP crossbar, 256, 1024, and 4096 columns are used in Figure 8b, c, and d, respectively. It is known that having more SP columns can result in a better recognition rate [12]. This is because each SP column can store a specific feature of tested vectors. If the number of SP columns becomes larger, then more features can be stored in the columns. Thereby, the recognition rate for the tested images can be improved with increasing the number of SP columns. The number of SP columns = 256 is the same condition for the memristor-implemented Convolutional Neural Network, where the testing image has 20 × 20 pixels, the kernel size is 5 × 5, and the number of kernels = 1. Similarly, the number of SP columns = 1024 is the same condition of Convolutional Neural Network, with 20 × 20 image, 5 × 5 kernel, and the number of kernels = 4. The number of SP columns = 4096 is the same condition of Convolutional Neural Network, with 20 × 20 image, 5 × 5 kernel, and the number of kernels = 16. In this simulation, we did not simulate the crossbars with SP columns more than 4096, because we do not use the number of kernels more than 16 for recognizing the MNSIT vectors, in Convolutional Neural Network.

Figure 8b shows MNIST recognition rate of the memristor crossbar with SP columns = 256. Here, the percentage of defects in the crossbar is changed from 0% to 20%. The percentage σ of memristance variation of HRS and LRS is assumed to be zero. For the percentage of defects = 0%, the crossbars without and with the boost-factor adjustment show the recognition rates of 77.3% and 77.6%, respectively. When the percentage of defects is very small, the boost-factor adjustment affects the recognition rate very little. However, if the percentage increases, the boost-factor adjustment plays an important role to keep the recognition rate as high as the rate of defects = 0%, as shown in Figure 8b. For the defects = 20%, the boost-factor adjustment can show the recognition rate better by as much as 30.6%, compared to the crossbar without the boost-factor adjustment.

Figure 8c is for the SP columns = 1024. As mentioned earlier, the crossbar with the SP columns = 1024 recognizes MNIST vectors better than the SP columns = 256. For the percentage of defects = 0%, the recognition rates of 256 and 1024 SP columns are 77.6% and 92.5%, respectively. As indicated in Figure 8b, the boost-factor adjustment in Figure 8c can maintain this good recognition rate, even though the percentage of defects is increased to 20%. For the percentage = 20%, the gap of recognition rates without and with the boost-factor adjustment is as much as 35.9%.

Figure 8d is for the SP columns = 4096. If the percentage of defects is 0%, the recognition rate of the crossbar is as high as 96.2%. In spite of the percentage of defects = 20%, the boost-factor adjustment can keep the rate as high as 94%, whereas the crossbar without the boost-factor adjustment is as low as 78%.

**Figure 9:**
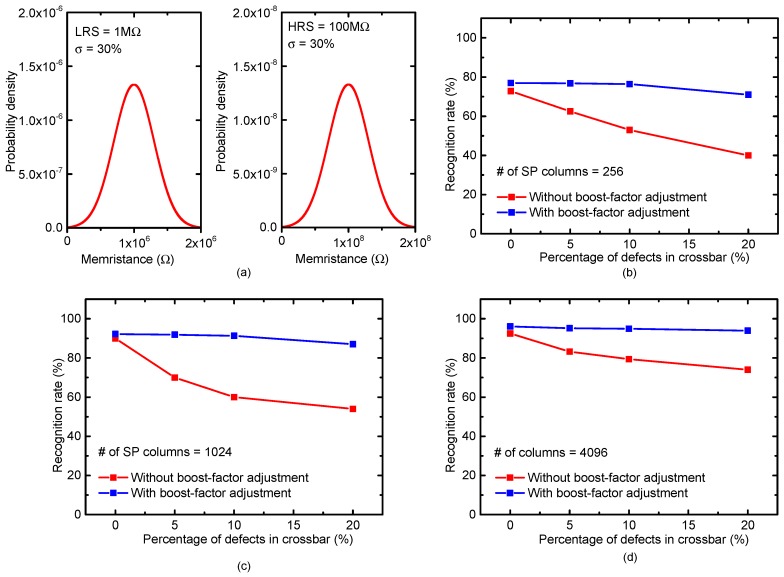
(**a**) The statistical distributions of LRS and HRS with the percentage σ of memristance variation = 30% in the simulation; (**b**) the MNIST recognition rate of the non-ideal crossbar with 256 SP columns and the percentage σ of memristance variation = 30%. Here, SP means Spatial Pooler.; (**c**) the MNIST recognition rate of the non-ideal crossbar with 1024 SP columns and the percentage σ of memristance variation = 30%; and (**d**) the MNIST recognition rate of the non-ideal crossbar with 4096 SP columns and the percentage σ of memristance variation = 30%.

Figure 9a shows the statistical distributions of HRS and LRS, where the percentage σ of memristance variation is assumed to be 30%. Figure 9 b, c, and d are for the SP columns = 256, 1024, and 4096, respectively. As indicated in Figure 9, the more SP columns can result in the better recognition rate. In Figure 9b, the percentage of defects is changed from 0% to 20%. For the percentage of defects = 0%, the boost-factor adjustment affects the recognition rate very little. However, if the percentage of defects is increased to 20%, the boost-factor adjustment can improve the recognition rate significantly compared to the crossbar without the boost-factor adjustment. Similarly, in Figure 9c with the SP columns = 1024, the recognition rates without and with the boost-factor adjustment are 54% and 87%, respectively, when the defects = 20%. In Figure 9d with the SP columns = 4096, the recognition rates without and with the boost-factor adjustment are 74% and 93.9%, respectively, when the defects = 20%.

## 4. Discussion

In this session, to understand the benefit of the proposed circuit exactly, we discuss and compare the following three SP schemes in Table 1: (1) Spatial-pooling without both the boost-factor adjustment and the defect-aware mapping, (2) spatial-pooling with the defect-aware mapping, and (3) spatial-pooling with the boost-factor adjustment.

First, we discuss the possibility of hardware implementation in Table 1. As mentioned earlier, (1) and (3) can be implemented in hardware. However, the defect-aware mapping of (2), as indicated in Figure 2d, demands very complicated circuits such as memory, processor, controller, etc. 

Second, the energy consumptions of the crossbar programming are compared among (1), (2), and (3) in Table 1. The amount of programming energy is simulated during the training time of 10,000 MNIST vectors (1) and (2) consume 3.9 mJ for programming the crossbar with HRS and LRS, according to Hebbian learning rule, as explained in Figure 4a. The energy overhead due to the boost-factor adjustment is less than ~0.05% of the crossbar programming energy. This is because each column has only one memristor for the boost-factor adjustment, compared to 400 cells per column for Hebbian learning. 

For the recognition rate, in Table 1 (1), without the boost-factor adjustment and defect-aware mapping, shows MNIST recognition rates of 77.3% and 55.6%, when the defects = 0% and 10%, respectively. Similarly, (2), with only the defect-aware mapping, shows the rates of 77.3% and 56.3%, when the defects = 0% and 10%, respectively. Without the boost-factor adjustment, the defective columns necessarily become activated frequently. The frequent activation of defective columns degrades the recognition rate significantly, as shown in (2) in Table 1. On the contrary, (3) with the boost-factor adjustment shows the rates of 77.6% and 77%, when the defects = 0% and 10%, respectively. It has very little loss of the recognition rate, in spite of the defects = 10%. The gap between the defects = 0% and 10% is negligibly small for the crossbar with the boost-factor adjustment.

We now discuss the relationship of this work to the previous works performed in HTM hardware realization. Actually, as a previous works of this paper, we developed the memristor crossbar circuits for performing the SP and TM operations of HTM, respectively [12,13]. However, in the previous works, we did not consider the memristor defects, which should be taken into account in the real memristor crossbar having defects of stuck-at-faults and variations. Thus, the SP hardware implemented with the real defective memristor crossbar can be an essential part of future HTM’s hardware system. Additionally, as a further work, we try to fabricate the crossbar having more than 100 memristors and combine the fabricated crossbar with the CMOS circuit to verify the SP operation by hardware, for testing the MNSIT vectors.

Finally, we discuss possible applications of the memristor-CMOS hybrid circuit of HTM’s hardware. As Internet of Things (IoT) sensors become more popular in human life and environment, an amount of data generated from the sensors becomes enormous [28,29,30]. To handle this huge amount of data from the physical world, we can think of the integration of IoT sensors and memristor-CMOS hybrid circuit into one chip [31,32]. By doing so, the unstructured data from the sensors can be pre-processed and interpreted near the sensors by the integrated memristor-CMOS hybrid circuit of HTM hardware. If we deliver all the data generated from the IoT sensors to the cloud, without any pre-processing of the unstructured data near the IoT sensors, an amount of computing energy demanded at the cloud may be huge [33]. Thus, the memristor-CMOS hybrid circuit that can perform the pre-processing of the unstructured data from the IoT sensors can be very useful for energy-efficient computing in future.

## 5. Conclusions

The SP of HTM has been known as the software framework to model human brain’s neocortical operation such as recognition, cognition, etc. However, mimicking the brain’s neocortical operation by hardware rather than software is more desirable, because the hardware not only describes the neocortical operation, but also employs the brain’s architectural advantages such as high energy efficiency, extreme parallel-computation, etc. 

To realize HTM’s SP by hardware, in this paper, we developed the memristor-CMOS hybrid circuit. One thing important for hardware implementation is that memristor defects such as stuck-at-faults, memristance variations, etc., should be considered in developing the memristor-CMOS hybrid circuit of SP. 

For considering memristor defects in hardware implementation, first, we showed that the boost-factor adjustment can make HTM’s SP defect-tolerant, because the false activation of defective columns can be suppressed. Second, we proposed the memristor-CMOS hybrid circuit with the boost-factor adjustment for realizing the defect-tolerant spatial-pooling in hardware. The proposed circuit does not rely on the conventional defect-aware mapping scheme, which cannot avoid the false activation of defective columns in spatial-pooling. For the MNIST data-set, the boost-factor adjusted crossbar with the defects = 10% was verified to have a rate loss as low as ~0.6%, compared to the ideal crossbar with the defects = 0%. On the contrary, the defect-aware mapping without the boost-factor adjustment demonstrated a significant rate loss, as much as ~21.0%. The energy overhead of the boost-factor adjustment was estimated to be as little as ~0.05% of the programming energy of the memristor synapse crossbar.

## Figures and Tables

**Table 1:** Comparison of possibility of hardware implementation, energy consumption for the crossbar programming, and MNIST recognition rate for the three SP schemes. Here, SP means Spatial Pooler. Energy consumption is calculated during the training time of 10,000 MNIST vectors.

	Possibility for Hardware Implementation	Energy Consumption of the Crossbar Programming (SP Column = 256)	MNIST Recognition Rate		
			# of SP Columns	Rate (%) Defects = 0%	Rate (%) Defects = 10%
(1) Spatial-pooling without the boost-factor adjustment and the defect-aware mapping	Able to be implemented with hardware	3.9 mJ for the crossbar programming	256	77.3	55.6
			1024	92	65.4
			4096	95.7	81.1
(2) Spatial-pooling with the defect-aware mapping	The defect-aware mapping in Figure 2d demands the very complicated hardware of memory, processor, controller, etc.	3.9mJ for the crossbar programming	256	77.3	56.3
			1024	92	66.5
			4096	95.7	82.4
(3) Spatial- pooling with the boost-factor adjustment	Able to be implemented with hardware	3.9 mJ for the crossbar programming, +2uJ for the boost-factor adjustment (Energy overhead due to boost-factor adjustment: ~0.05%)	256	77.6	77
			1024	92.5	91.8
			4096	96.2	95.4
